# MTG16 regulates colonic epithelial differentiation, colitis, and tumorigenesis by repressing E protein transcription factors

**DOI:** 10.1172/jci.insight.153045

**Published:** 2022-05-23

**Authors:** Rachel E. Brown, Justin Jacobse, Shruti A. Anant, Koral M. Blunt, Bob Chen, Paige N. Vega, Chase T. Jones, Jennifer M. Pilat, Frank Revetta, Aidan H. Gorby, Kristy R. Stengel, Yash A. Choksi, Kimmo Palin, M. Blanca Piazuelo, Mary Kay Washington, Ken S. Lau, Jeremy A. Goettel, Scott W. Hiebert, Sarah P. Short, Christopher S. Williams

**Affiliations:** 1 Program in Cancer Biology and; 2 Medical Scientist Training Program, Vanderbilt University School of Medicine, Nashville, Tennessee, USA.; 3 Department of Pathology, Microbiology and Immunology, Vanderbilt University, Nashville, Tennessee, USA.; 4 Willem Alexander Children’s Hospital, Leiden University Medical Center, Leiden, Netherlands.; 5 Department of Medicine, Division of Gastroenterology, Hepatology, and Nutrition, Vanderbilt University Medical Center, Nashville, Tennessee, USA.; 6 Vanderbilt University, Nashville, Tennessee, USA.; 7 Program in Chemical and Physical Biology, Vanderbilt University School of Medicine, Nashville, Tennessee, USA.; 8 Epithelial Biology Center, Vanderbilt University Medical Center, Nashville, Tennessee, USA.; 9 Department of Cell and Developmental Biology and; 10 Department of Biochemistry, Vanderbilt University School of Medicine, Nashville, Tennessee, USA.; 11 Veterans Affairs Tennessee Valley Health Care System, Nashville, Tennessee, USA.; 12 Center for Mucosal Inflammation and Cancer, Vanderbilt University Medical Center, Nashville, Tennessee, USA.; 13 Department of Medical and Clinical Genetics,; 14 Applied Tumor Genomics Research Program, Research Programs Unit, and; 15 iCAN Digital Precision Cancer Medicine Flagship, University of Helsinki, Helsinki, Finland.; 16 Vanderbilt-Ingram Cancer Center, Vanderbilt University Medical Center, Nashville, Tennessee, USA.

**Keywords:** Cell Biology, Gastroenterology, Inflammatory bowel disease

## Abstract

Aberrant epithelial differentiation and regeneration contribute to colon pathologies, including inflammatory bowel disease (IBD) and colitis-associated cancer (CAC). Myeloid translocation gene 16 (MTG16, also known as CBFA2T3) is a transcriptional corepressor expressed in the colonic epithelium. MTG16 deficiency in mice exacerbates colitis and increases tumor burden in CAC, though the underlying mechanisms remain unclear. Here, we identified MTG16 as a central mediator of epithelial differentiation, promoting goblet and restraining enteroendocrine cell development in homeostasis and enabling regeneration following dextran sulfate sodium–induced (DSS-induced) colitis. Transcriptomic analyses implicated increased Ephrussi box–binding transcription factor (E protein) activity in MTG16-deficient colon crypts. Using a mouse model with a point mutation that attenuates MTG16:E protein interactions (*Mtg16*^P209T^), we showed that MTG16 exerts control over colonic epithelial differentiation and regeneration by repressing E protein–mediated transcription. Mimicking murine colitis, *MTG16* expression was increased in biopsies from patients with active IBD compared with unaffected controls. Finally, uncoupling MTG16:E protein interactions partially phenocopied the enhanced tumorigenicity of *Mtg16^–/–^* colon in the azoxymethane/DSS-induced model of CAC, indicating that MTG16 protects from tumorigenesis through additional mechanisms. Collectively, our results demonstrate that MTG16, via its repression of E protein targets, is a key regulator of cell fate decisions during colon homeostasis, colitis, and cancer.

## Graphical abstract

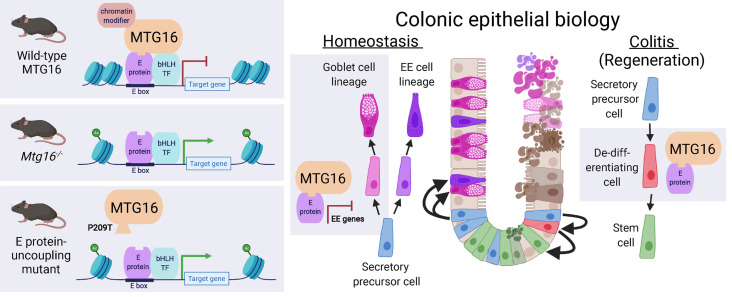

## Introduction

The colonic epithelium is a complex, self-renewing tissue composed of specialized cell types with diverse functions (1). Stem cells at the base of the colon crypt divide and differentiate into absorptive and secretory cells. Conversely, colonic epithelial regeneration in response to injury occurs through dedifferentiation of committed absorptive and secretory lineage cells (2–4). Secretory lineage dysregulation is implicated in inflammatory bowel disease (IBD), diabetes, and even behavioral changes (5–9), and aberrant regenerative programs may lead to uncontrolled cell proliferation and dysplasia (10). Thus, these processes must be tightly controlled by coordinated networks of transcriptional activators and repressors (1).

Myeloid translocation gene 16 (MTG16, also known as CBFA2T3) is a transcriptional corepressor that regulates cell fate decisions by bridging transcription factors and chromatin modifiers to repress transcription of target loci (11–14). Repression targets of MTG16 vary depending on the expression gradients of other components of the repression complex (14, 15). MTG16 represses stem cell genes and pan-secretory genes in the homeostatic small intestine (SI) (16). We have previously shown that MTG16 deficiency leads to increased injury in dextran sulfate sodium–induced (DSS-induced) colitis (17) and tumor burden in azoxymethane (AOM)/DSS-induced inflammatory carcinogenesis and that these were epithelium-specific phenotypes (18). However, the precise mechanisms by which MTG16 controls colonic homeostasis, regeneration, and tumorigenesis remain unknown.

One family of transcription factors MTG16 represses are class I basic helix-loop-helix (bHLH) transcription factors, also known as E proteins (14, 19, 20). E proteins, which consist of E12 and E47 (splice variants of *E2A*), HEB, and E2-2, bind to consensus Ephrussi box (E box) DNA sequences to activate transcription (20–22). Because E proteins are widely expressed, the specificity of their transcriptional targets is dictated through oligomerization with class II bHLH transcription factors, which are expressed with spatial and temporal specificity (23). E protein–mediated control of stem cell dynamics, lineage allocation, and differentiation has been most thoroughly investigated in the hematopoietic system (19, 23–29) and neurogenesis (23, 30–33), outside the colon.

In this study, we defined the topology of *Mtg16* expression in the colon and discovered functions of MTG16 in the colonic epithelium. We used an MTG16:E protein uncoupling point mutant mouse model to demonstrate that the mechanism driving these phenotypes was repression of E protein transcription factors by MTG16. We tested the functional impact of this regulatory relationship in murine models of IBD and colitis-associated cancer (CAC) and correlated our observations with patient data. Overall, we demonstrate potentially novel, context-specific roles for MTG16 in colonic epithelial lineage allocation and protection from colitis that depend on its repression of E protein–mediated transcription.

## Results

### MTG16 is expressed in goblet cells in human and murine colon.

**Figure 1:**
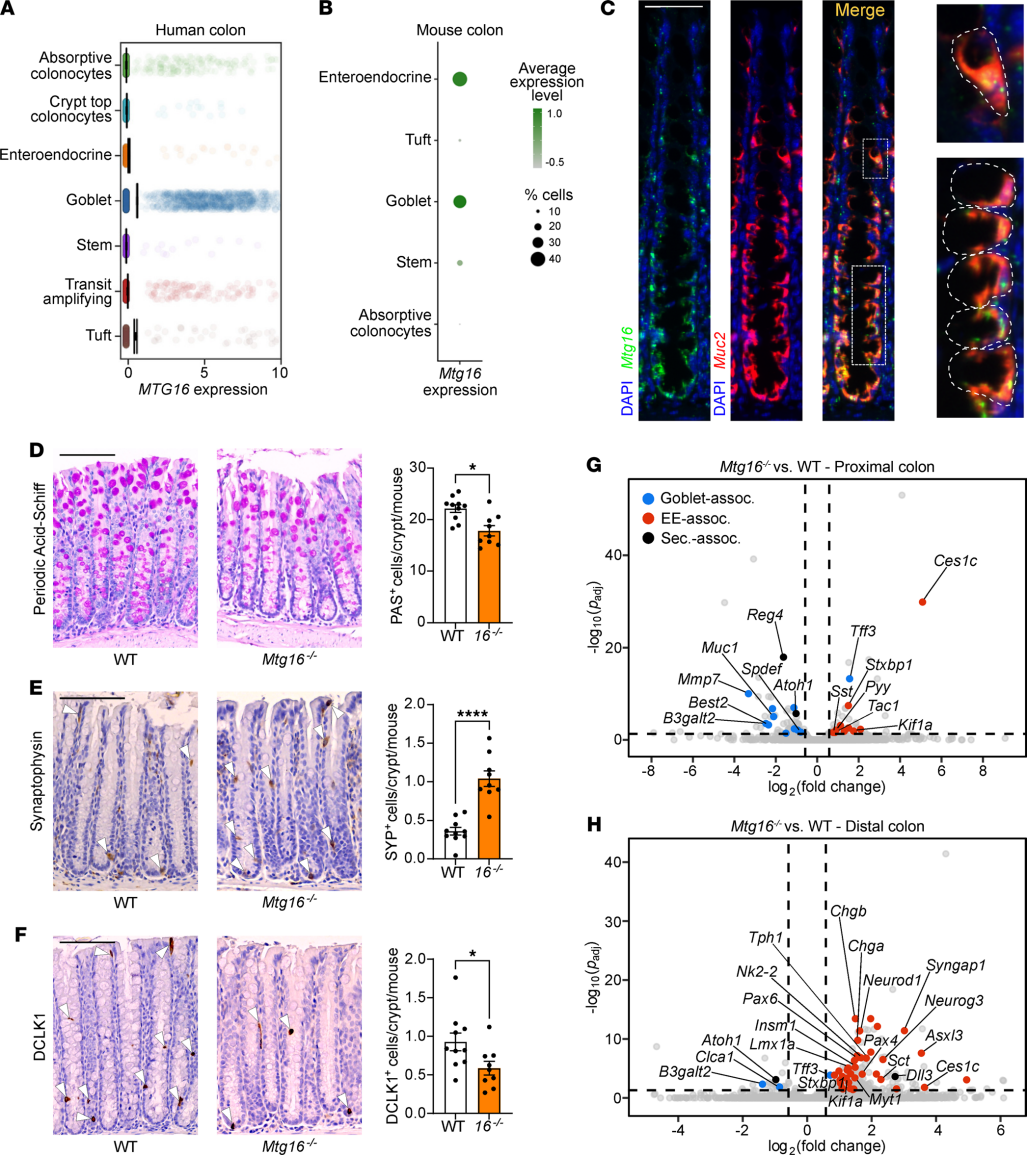
MTG16 is expressed in colonic secretory cells, and its deficiency alters secretory lineage allocation. (**A**) *MTG16* expression in cell populations generated from scRNA-Seq of normal human colon biopsies (discovery cohort, *n* = 35 samples, 30,374 cells) (validation cohort, Supplemental Figure 1A). (**B**) *Mtg16* expression in scRNA-Seq of WT mouse colon (*n* = 3 mice, 3653 cells). Color gradient represents average *Mtg16* expression level in each cell population. Dot size represents percentage of cells in each population expressing *Mtg16*. Uniform manifold approximation and projection plots are in Supplemental Figure 1B. (**C**) RNAscope in situ hybridization of *Mtg16* and *Muc2* in WT mouse colon. Scale bar = 50 μm. Goblet cells are outlined in the insets at right. (**D**–**F**) WT and *Mtg16^–/–^* mouse colon (*n* = 10 WT, 9 *Mtg16^–/–^*) stained for (**D**) goblet cells by PAS stain, (**E**) enteroendocrine cells by IHC for SYP, and (**F**) tuft cells by IHC for doublecortin-like kinase 1 (DCLK1). Representative images at left. SYP^+^ and DCLK1^+^ cells are indicated with white arrowheads. Scale bars = 100 μm. *P < 0.05, ****P < 0.0001, 2-tailed Mann-Whitney *U* test. (**G** and **H**) Volcano plots demonstrating differentially expressed genes in (**G**) proximal (*n* = 2 WT, 2 *Mtg16^–/–^*) and (**H**) distal (*n* = 4 WT, 4 *Mtg16^–/–^*) colonic epithelial isolates by RNA-Seq. Horizontal dashed line, *P*_adj_ < 0.05 by DESeq2. Vertical dashed lines, fold change = 1.5. Dot colors: goblet-associated (blue), enteroendocrine-associated (EE-associated) (red), and secretory-associated (black) genes.

To understand the topography of *MTG16* expression in the human colon, we queried single-cell RNA-Seq (scRNA-Seq) data sets generated from 2 independent cohorts of normal human colon samples (10). Interestingly, we observed strong *MTG16* expression in goblet cells (Figure 1A and Supplemental Figure 1A; supplemental material available online with this article; https://doi.org/10.1172/jci.insight.153045DS1). To understand if this expression pattern was evolutionarily conserved, we queried our inDrop scRNA-Seq generated from WT murine colon crypts (34, 35) and similarly found that *Mtg16* was expressed in goblet and enteroendocrine clusters (Figure 1B and Supplemental Figure 1B). RNAscope for *Mtg16* and *Muc2*, the predominant mucin expressed by colonic goblet cells (36), demonstrated strong colocalization in the murine colonic epithelium (Figure 1C).

### Loss of MTG16 distorts colonic secretory cell differentiation.

We next assessed secretory lineage allocation in the *Mtg16^–/–^* homeostatic colon epithelium, which had not previously been investigated to our knowledge (Figure 1, D–F). *Mtg16^–/–^* mice had fewer goblet and tuft cells but, unexpectedly, increased synaptophysin-positive (SYP^+^) enteroendocrine cells per colon crypt (Figure 1E). In contrast, and in agreement with prior studies (16, 37), *Mtg16^–/–^* mice had fewer goblet and tuft cells, but no increase in enteroendocrine cells, in the SI (Supplemental Figure 2). These data implicated a colon-specific role for MTG16 in orchestrating cell type allocation within the secretory lineage.

Prior studies have demonstrated subtle differences in secretory cell differentiation along the length of the colon (36, 38–41). Thus, we stratified our human scRNA-Seq data sets by proximal and distal colon and examined *MTG16* expression. *MTG16* was consistently expressed in goblet cells, but its expression pattern in other secretory cell types varied (Supplemental Figure 3). We next queried the *Tabula Muris* (42), a murine colon scRNA-Seq data set that specifies whether certain clusters originate from the proximal or distal colon, for *Mtg16* expression. In the proximal colon, *Mtg16* was enriched in all goblet cell clusters (Supplemental Figure 4, A and B). In the distal colon, *Mtg16* was enriched in early progenitor cells still expressing the stem cell marker *Lgr5* (*Lgr5*^+^, amplifying, undifferentiated cells) and most goblet cells, except those located at the top of the crypt (Supplemental Figure 4, A and B). Bulk RNA-Seq of proximal and distal WT colon revealed higher *Mtg16* expression in the proximal colon, potentially reflecting its higher goblet cell density (Supplemental Figure 4C). We confirmed this *Mtg16* expression pattern by RNAscope (Supplemental Figure 5).

**Figure 2:**
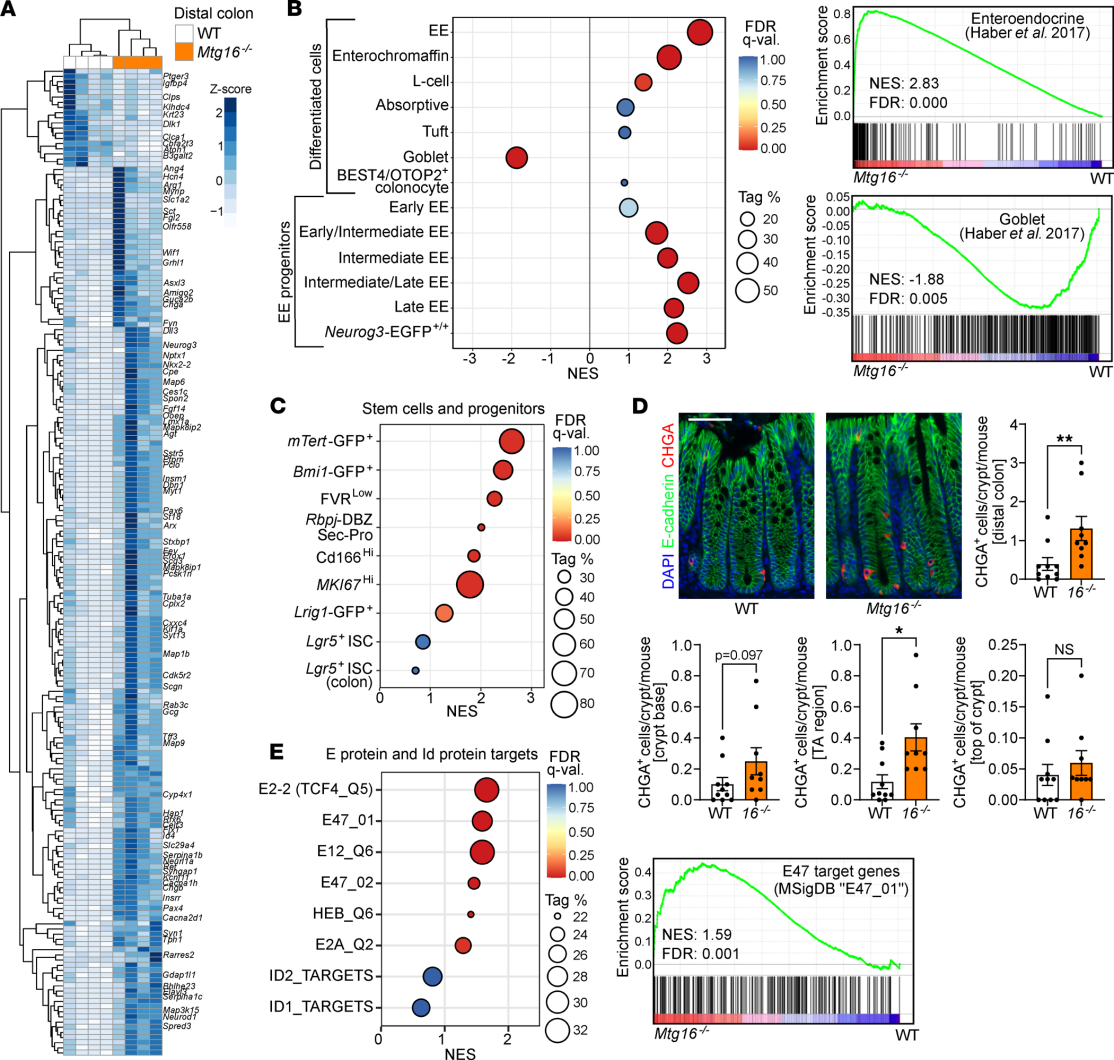
MTG16 deficiency is associated with increased enteroendocrine cell markers, reserve stem cell gene signatures, and E protein target upregulation in the distal colon. (**A**) Unsupervised hierarchical clustering of differentially expressed genes in *Mtg16^–/–^* versus WT (*n* = 4) distal colonic epithelial isolates by RNA-Seq. Gene expression in individual samples is displayed by *z* score (normalized count). Genes of interest are labeled at right. (**B**) GSEA of distal colon RNA-Seq using gene sets representing epithelial cell types (described in Supplemental Tables 3 and 4 and cited in the supplement). Individual GSEA plots of EE and goblet cell signatures are displayed at right. (**C**) GSEA of distal colon RNA-Seq using gene sets representing stem and other progenitor cell populations (Supplemental Tables 3 and 4). (**D**) Staining and quantification of CHGA^+^ cells/crypt (top) stratified by location along the crypt axis (bottom). Scale bar = 50 μm. *n* = 10 WT, 9 *Mtg16^–/–^*. *P < 0.05, **P < 0.01 by 2-tailed Mann-Whitney test. TA, transit amplifying. (**E**) GSEA of distal colon RNA-Seq using MSigDB (51) gene sets representing E protein and Id protein transcriptional signatures (Supplemental Table 3). Individual GSEA plot of the E47 target gene signature at right. NES, normalized enrichment score (ES); Tag %, the percentage of gene hits before (for positive ES) or after (for negative ES) the peak in the running ES, indicating the percentage of genes contributing to the ES. FDR *q* < 0.05 is considered significant.

Due to these differences, we performed separate bulk RNA-Seq analyses of *Mtg16^–/–^* proximal and distal colon epithelial isolates. In both colon segments, goblet-associated genes were downregulated, and enteroendocrine-associated genes were upregulated, compared with WT (Figure 1, G and H). However, goblet cell–associated gene downregulation was more pronounced in the proximal colon (Figure 1G), while enteroendocrine cell–associated gene upregulation was more striking in the distal colon (Figure 1H). Closer inspection of the distal *Mtg16^–/–^* colon revealed marked upregulation of class II bHLH transcription factors required for enteroendocrine cell differentiation (*Neurog3* and *Neurod1*) and enteroendocrine cell hormone products (chromogranin A, *Chga*; *Gcg*; *Sct*) (Figure 2A). We further investigated the distal colon using gene set enrichment analysis (GSEA) with custom gene sets derived from the literature (described in Supplemental Tables 3 and 4). GSEA of distal colon RNA-Seq demonstrated significant enrichment of all stages of enteroendocrine cells during differentiation except the earliest progenitor cells, in which expression of *Neurog3*, which is required for enteroendocrine cell differentiation (43–46), had not yet been induced (45) (Figure 2B). Concurrently, the goblet cell gene signature was significantly de-enriched in the absence of MTG16 (Figure 2B).

Notably, *Mtg16^–/–^* versus WT RNA-Seq did not show significant differences in the stem cell genes *Lgr5* and *Ascl2*, distinct from *Mtg16^–/–^* SI (16). Additionally, GSEA using gene sets representing stem cells and other early progenitors (Supplemental Tables 3 and 4) did not demonstrate enrichment of any gene sets representing *Lgr5*^+^ crypt base columnar stem cells (CBCs) (Figure 2C and Supplemental Figure 6A). We did observe enrichment of gene sets representing *Bmi1*^+^ and *mTert*^+^ “reserve” stem cells (Figure 2C), recently shown to be enteroendocrine cells expressing *Chga* (47). Although CHGA^+^ cells were increased overall, they were not localized to the crypt base (including the +4/+5 cell position) (Figure 2D). Altogether, these data indicate that MTG16 promotes goblet cell differentiation, at the expense of enteroendocrine cell differentiation, during colonic secretory lineage allocation.

### MTG16-driven goblet cell differentiation is dependent on its repression of E protein–mediated transcription.

We next sought to illuminate potential mechanisms by which MTG16 promotes the goblet lineage and restricts the enteroendocrine lineage. We and others have previously shown that MTG16 negatively regulates major transcription factor effectors of the WNT and Notch pathways in the hematopoietic system (15, 48) and SI (16). Our RNA-Seq revealed upregulation of the Notch ligand *Dll3* (Figure 1H and Figure 2A). However, several WNT pathway inhibitors (*Wif1*, *Cxxc4*) and the Notch pathway inhibitor *Neurl1a* were also significantly upregulated (Figure 2A). We thus performed GSEA for the canonical WNT and Notch pathways, as well as other pathways important in colonic epithelial homeostasis and differentiation (1). GSEA did not identify global canonical WNT or Notch pathway enrichment or de-enrichment in *Mtg16^–/–^* homeostatic colon crypts (Supplemental Figure 6B). We next investigated whether the target genes of known MTG16-interacting transcription factors, including E proteins (14, 19, 20), were dysregulated in *Mtg16^–/–^* colon crypts. As stated previously, E proteins coordinate lineage specification and differentiation by interacting with cell type–specific class II bHLH transcription factors (21). The E proteins E12, E47, and HEB are known to be expressed in SI crypts and adenomas (49), and E12 and E47 bind to NEUROD1 to induce transcription of secretin in the duodenum (50). Here, in *Mtg16^–/–^* colon crypts, we observed significant enrichment of all E protein gene signatures available in the Molecular Signatures Database (MSigDB) (51) (Figure 2E and Supplemental Table 3). Gene sets associated with upregulation of Id1 and Id2, proteins that counteract E protein activity, were not enriched (Figure 2E). These data demonstrated a strong induction of E protein–mediated transcription in the absence of repression by MTG16. Thus, we hypothesized that MTG16 drives goblet cell differentiation from secretory progenitors by inhibiting E protein–mediated transcription of key enteroendocrine genes.

**Figure 3:**
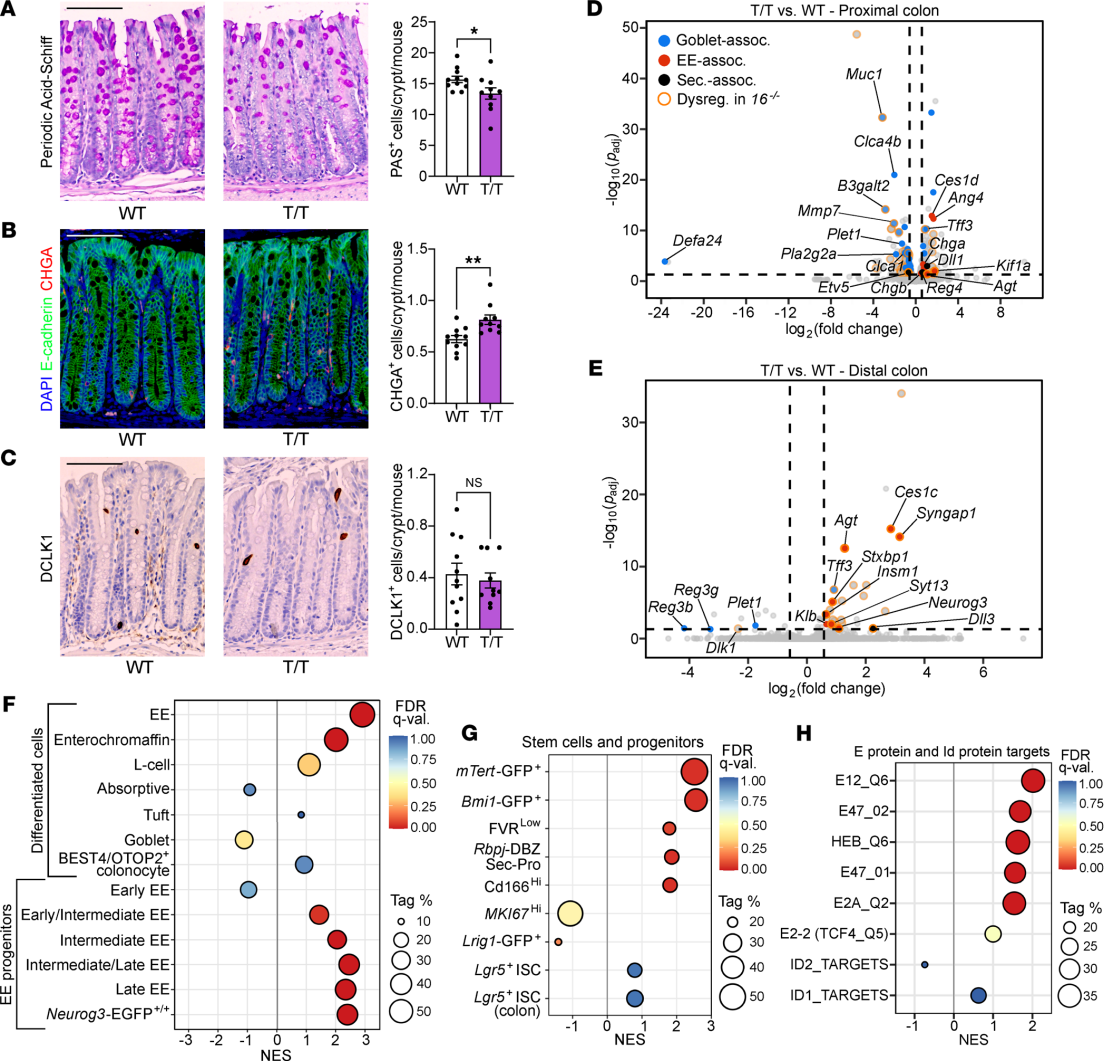
MTG16-driven colonic epithelial lineage allocation is dependent on its repression of the E protein transcription factors E2A and HEB. (**A**) WT and *Mtg16^T/T^* (T/T) mouse colon (*n* = 11 WT, 10 *Mtg16^T/T^*) stained for (**A**) goblet cells by PAS stain, (**B**) EE cells by immunofluorescence staining for CHGA, and (**C**) tuft cells by IHC for DCLK1. Representative images at left. Scale bars = 100 μm. *P < 0.05, **P < 0.01 by 2-tailed Mann-Whitney test. (**D** and **E**) Volcano plots demonstrating differentially expressed genes in (**D**) proximal (*n* = 2 WT, 2 *Mtg16^T/T^*) and (**E**) distal (*n* = 3 WT, 4 *Mtg16^T/T^*) colonic epithelial isolates by RNA-Seq. Horizontal dashed line indicates *P*_adj_ < 0.05 by DESeq2. Vertical dashed lines indicate fold change = 1.5. Dot colors indicate goblet-associated (blue), EE-associated (red), and secretory-associated (black) genes. Dots outlined in orange represent genes that are upregulated or downregulated in the same direction in the corresponding *Mtg16^–/–^* colon segment (Dysreg. in *16^–/–^*). (**F**–**H**) GSEA of distal colon RNA-Seq using gene sets representing (**F** and **G**) epithelial cell types and (**H**) E protein transcriptional signatures (Supplemental Tables 3 and 4). Tag %, the percentage of gene hits before (for positive ES) or after (for negative ES) the peak in the running ES, indicating the percentage of genes contributing to the ES. FDR *q* < 0.05 is considered significant.

We next tested this hypothesis by selectively modulating MTG16-mediated repression of E protein transcriptional activity in vivo. To do so, we CRISPR-generated a mouse with a point mutation resulting in a proline-to-threonine substitution in MTG16 (*Mtg16*^P209T^) that attenuates MTG16:E2A and MTG16:HEB binding and functionally reduces MTG16-mediated repression of E protein transcriptional activity (52) (Supplemental Figure 7; full, uncropped gels available in online supplemental material). Similar to *Mtg16^–/–^* mice, homozygous *Mtg16*^P209T^ mutant (*Mtg16^T/T^*) mice had fewer periodic acid–Schiff–positive (PAS^+^) goblet cells and more CHGA^+^ enteroendocrine cells per crypt compared with littermate-matched WT controls, though tuft cell frequency per crypt was unchanged (Figure 3, A–C). We next performed bulk RNA-Seq on *Mtg16^T/T^* proximal and distal colon crypt isolates. In both colon segments, goblet cell genes were downregulated compared with WT, while enteroendocrine cell genes were upregulated compared with WT, in a pattern resembling that of *Mtg16^–/–^* colon (Figure 3, D and E). Notably, the distal colon exhibited significant upregulation of *Neurog3* (Figure 3E). Additionally, GSEA of the overall enteroendocrine signature, key enteroendocrine progenitor genes, and *Bmi1*^+^ and *mTert*^+^ stem cell signatures all largely phenocopied the *Mtg16^–/–^* distal colon (Figure 3, F and G, and Supplemental Tables 3 and 4). Confirming that the MTG16^P209T^ mutation functionally results in increased E protein–mediated transcription in the colonic epithelium, the gene sets representing E2A and HEB transcriptional targets were also enriched in *Mtg16^T/T^* colonic epithelial isolates (Figure 3H).

**Figure 4:**
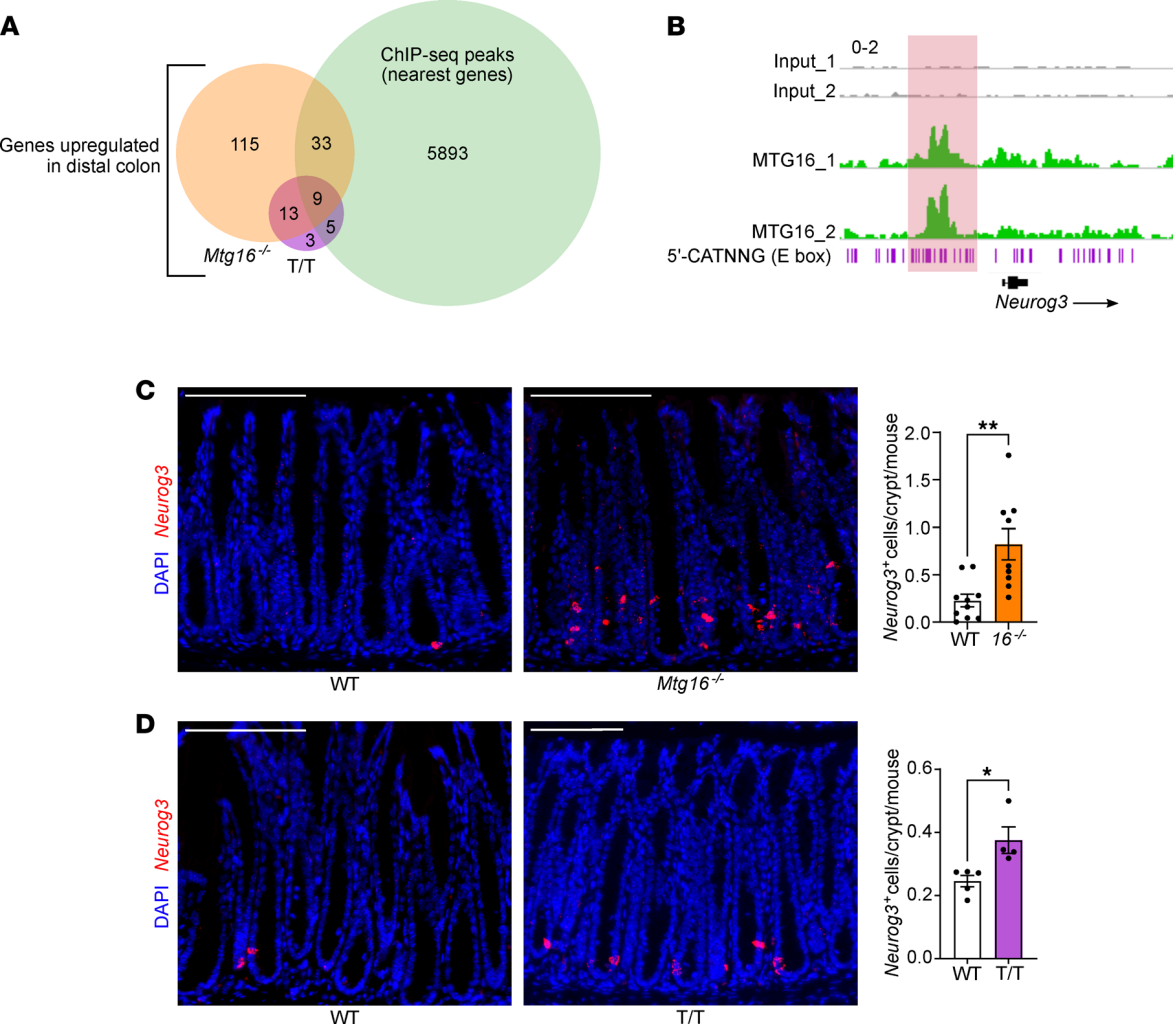
MTG16 represses E protein–mediated transcription of *Neurog3*. (**A**) Venn diagram of upregulated (*P*_adj_ < 0.05) genes from bulk RNA-Seq of WT versus *Mtg16^–/–^* (orange) and WT versus *Mtg16^T/T^* (T/T) (purple) distal colonic crypt isolates (*n* = 3–4) and genes with TSSs nearest to MTG16 peaks in a ChIP-Seq data set generated by Baulies et al. (16) (green). Table 1 provides more information about the common upregulated genes. (**B**) MTG16 occupancy of an E box (5′-CATNNG)-rich region 5′ to *Neurog3* in ChIP-Seq by Baulies et al. (16). Scale: 0–2. (**C** and **D**) Staining and quantification of *Neurog3*^+^ EE progenitor cells by RNAscope in (**C**) WT versus *Mtg16^–/–^* colon (*n* = 10 WT and 9 *Mtg16^–/–^*) and (**D**) WT versus *Mtg16^T/T^* colon (*n* = 5 WT and 4 *Mtg16^T/T^*). *P < 0.05, **P < 0.01 by 2-tailed Mann-Whitney test. Scale bars = 100 μm.

We next compared RNA-Seq results from *Mtg16^–/–^* versus WT and *Mtg16^T/T^* versus WT distal colon crypts and observed that 22 genes were upregulated in both data sets (Figure 4A and Table 1). Many of these genes (e.g., *Neurog3*, *Insm1*, *Insrr*, *Pex5l*, *Syt13*, *Syngap1*, *Kcnh6*, and *Agt*) were enteroendocrine associated (Table 1). A publicly available MTG16 ChIP-Seq data set from SI crypts (16) exhibited significant MTG16 occupancy proximal to 11 of the 22 genes (Table 1), including a large peak at an E box–rich site upstream of *Neurog3* (Figure 4B). Additionally, *Neurog3^+^* enteroendocrine progenitor cells were markedly increased in both *Mtg16^–/–^* and *Mtg16^T/T^* colon crypts as assessed by RNAscope (Figure 4, C and D). Altogether, these data indicated an MTG16:E protein–dependent mechanism of colonic secretory lineage allocation, probably via inhibition of NEUROG3-mediated commitment to the enteroendocrine cell lineage.

### MTG16 is upregulated in patients with active IBD.

**Figure 5:**
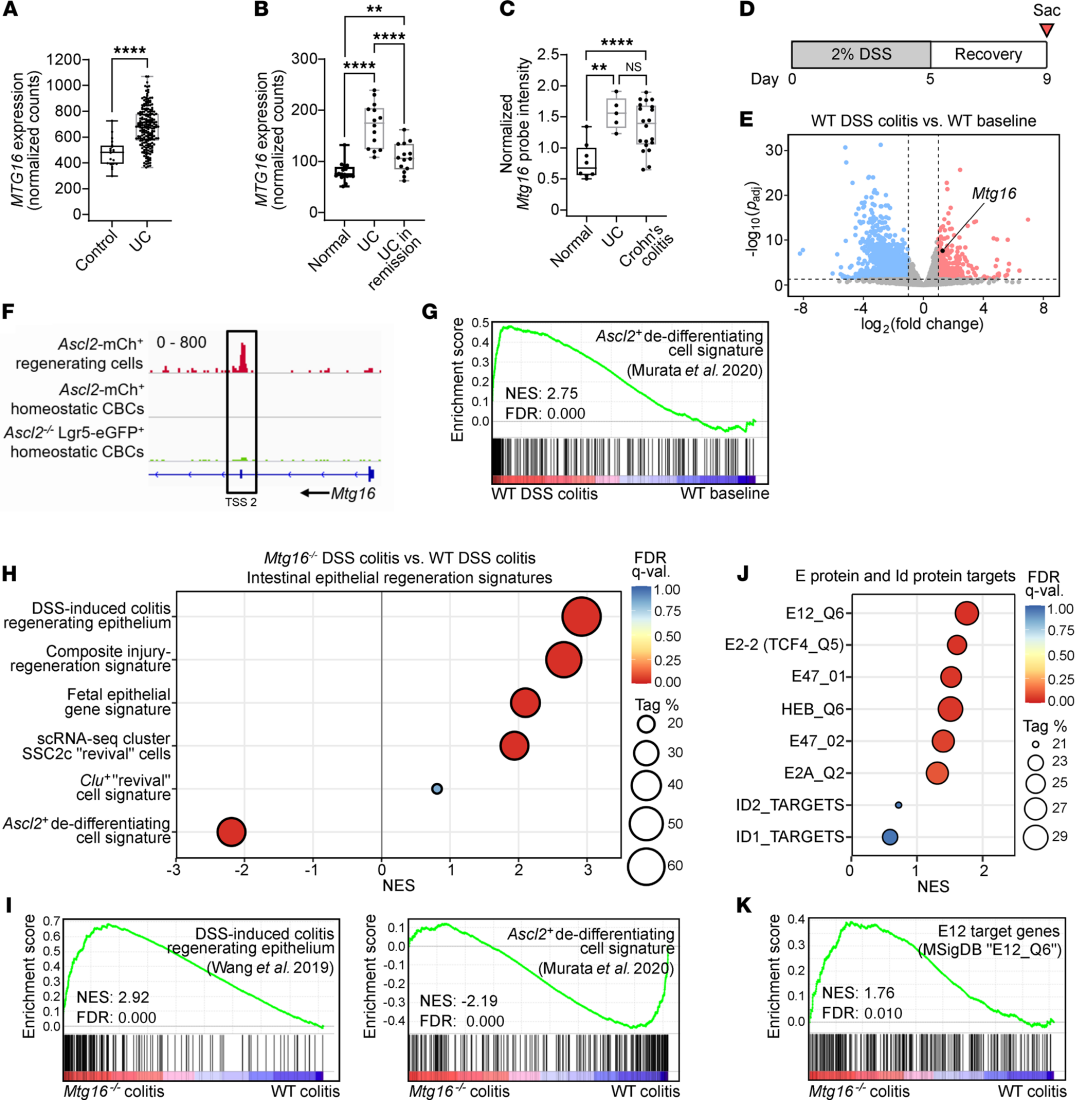
*Mtg16* is upregulated in experimental colitis and patients with IBD and is required for ASCL2-mediated colonic epithelial regeneration. (**A**) *MTG16* expression from an RNA-Seq data set generated from rectal biopsies of treatment-naive pediatric UC patients (*n* = 206) versus unaffected controls (*n* = 20) in the PROTECT study (58). Transcript counts were normalized by DESeq2. ****P_adj_ < 0.0001 by DESeq2. (**B**) *MTG16* expression in an RNA-Seq data set from adult UC patients with active disease (*n* = 14), UC patients in remission (*n* = 14), and unaffected control patients (*n* = 16) (60). Transcript counts were normalized by DESeq2. **P_adj_ < 0.01 and ****P_adj_ < 0.0001 by pairwise DESeq2. (**C**) *MTG16* expression in a microarray data set comparing UC (*n* = 5), Crohn’s colitis (*n* = 20), and unaffected (*n* = 8) patient biopsies (61). **P_adj_ < 0.01 and ****P_adj_ < 0.0001 by limma in GEO2R. Normalized probe intensity is plotted for data visualization. (**A**–**C**) The lines within each box represent the mean, the bounds of the boxes represent 25th to 75th percentiles, and the bounds of the whiskers represent the range of the data. All data points are shown. (**D**) Schematic of the DSS injury-regeneration model in which mice are treated with 2% for 5 days followed by 4 days of recovery. (**E**) Volcano plot demonstrating significantly downregulated (blue) and upregulated (red) genes in distal colon crypt isolates from WT mice following DSS-induced injury and regeneration compared with baseline (*n* = 3). Horizontal dashed line indicates *P*_adj_ < 0.05 by DESeq2. Vertical dashed lines indicate fold change = 2. (**F**) ASCL2 occupancy near an *Mtg16* transcription start site (TSS 2) in a ChIP-Seq data set of FACS-sorted *Ascl2*^+^ regenerating cells compared to *Lgr5^+^* homeostatic CBCs generated by Murata et al. (2). Scale: 0–800. (**G**) GSEA plot demonstrating significant enrichment of the *Ascl2*^+^ dedifferentiating cell signature (Supplemental Tables 3 and 4 and citation in the supplement) in distal colon crypt isolates from WT mice following DSS-induced colitis versus WT mice at baseline (*n* = 3). (**H**–**K**) GSEA performed on RNA-Seq of *Mtg16^–/–^* versus WT distal colon crypt isolates following DSS injury-regeneration (*n* = 3 WT, 4 *Mtg16^–/–^*) using (**H** and **I**) epithelial regeneration-associated gene sets and (**J** and **K**) gene sets representing E protein transcriptional signatures (Supplemental Tables 3 and 4 and citations in the supplement). (**G**–**K**) Tag %, the percentage of gene hits before (for positive ES) or after (for negative ES) the peak in the running ES, indicating the percentage of genes contributing to the ES. FDR *q* < 0.05 is considered significant.

There has been considerable interest in the role of secretory cell lineage misallocation and dysfunction in the pathogenesis of IBD, which includes ulcerative colitis (UC) and Crohn’s disease and is defined by continuous epithelial injury and regeneration (8, 36, 53–57). Additionally, several large RNA-Seq studies of human UC biopsies have recently been published, including the Predicting Responses to Standardized Pediatric Colitis Therapy (PROTECT) study (58, 59) and mucosal biopsies from patients with active UC, patients with UC in remission, and healthy controls (60). We found that *MTG16* was upregulated in patients with UC in both RNA-Seq data sets (Figure 5, A and B). *MTG16* expression decreased with remission yet remained elevated compared with unaffected controls (Figure 5B). Last, a microarray data set from pediatric colon biopsies (61) demonstrated upregulation of *MTG16* in both UC and Crohn’s colitis (Figure 5C). Collectively, these data show that increased *MTG16* expression is correlated with active injury and regeneration in IBD.

### MTG16 contributes to colon crypt regeneration following DSS-induced colitis.

As stated previously, MTG16 deficiency increases disease severity in DSS-induced colitis (17), a mouse model of IBD (schematic in Figure 5D) that recapitulates histologic features of human UC (62). We first assessed *Mtg16* expression in DSS-induced colitis to determine whether it would recapitulate the increased *MTG16* expression in human IBD. Because DSS treatment largely affects the distal colon (62), we compared distal WT colon crypt epithelial isolates after DSS-induced injury and regeneration with WT distal colon crypts at baseline. Indeed, *Mtg16* expression was significantly increased in regenerating colon crypts (Figure 5E), similar to human IBD. Thus, we posited that MTG16 expression may be critical for epithelial regeneration in colitis.

The cellular sources of intestinal repair have been a major focus of the field over the last several decades. Recently, Murata et al. demonstrated that any CBC progeny cell can dedifferentiate and repopulate ablated CBCs by inducing expression of *Ascl2* (2). After CBC ablation, 238 significantly upregulated genes defined the dedifferentiating ASCL2^+^ cells compared with resting CBCs. Interestingly, *Mtg16* was one of these few significantly upregulated genes (log_2_[fold change] = 2.05, *P*_adj_ = 0.004) (2). Furthermore, ASCL2 ChIP-Seq data in the dedifferentiating population (2) yielded a peak at a second TSS of *Mtg16*, suggesting that ASCL2 induces *Mtg16* expression during colonic epithelial regeneration (Figure 5F).

To confirm that the ASCL2-driven regeneration program described by Murata et al. was relevant to crypt regeneration following DSS-induced injury, we constructed a gene set consisting of the 238 genes upregulated in *Ascl2*^+^ regenerating cells (2) (Supplemental Tables 3 and 4), performed GSEA, and observed significant enrichment of this signature in WT distal colon crypts after DSS-induced regeneration (Figure 5G). Conversely, this regeneration signature was significantly de-enriched in *Mtg16^–/–^* colon crypts following DSS-induced injury, despite enrichment of other gene signatures associated with intestinal epithelial regeneration (Figure 5, H and I, and Supplemental Tables 3 and 4) (63–67). Together, these data implicate MTG16 as a component of ASCL2-driven regeneration and repair in the colon. Similar to our findings in colon homeostasis, E protein transcriptional signatures were enriched in post-DSS *Mtg16^–/–^* colon by GSEA (Figure 5, J and K), leading us to hypothesize that the contribution of MTG16 to colon crypt regeneration occurs via repression of E protein–mediated transcription.

### Attenuation of MTG16:E protein interactions disables epithelial regeneration following DSS-induced colitis.

**Figure 6:**
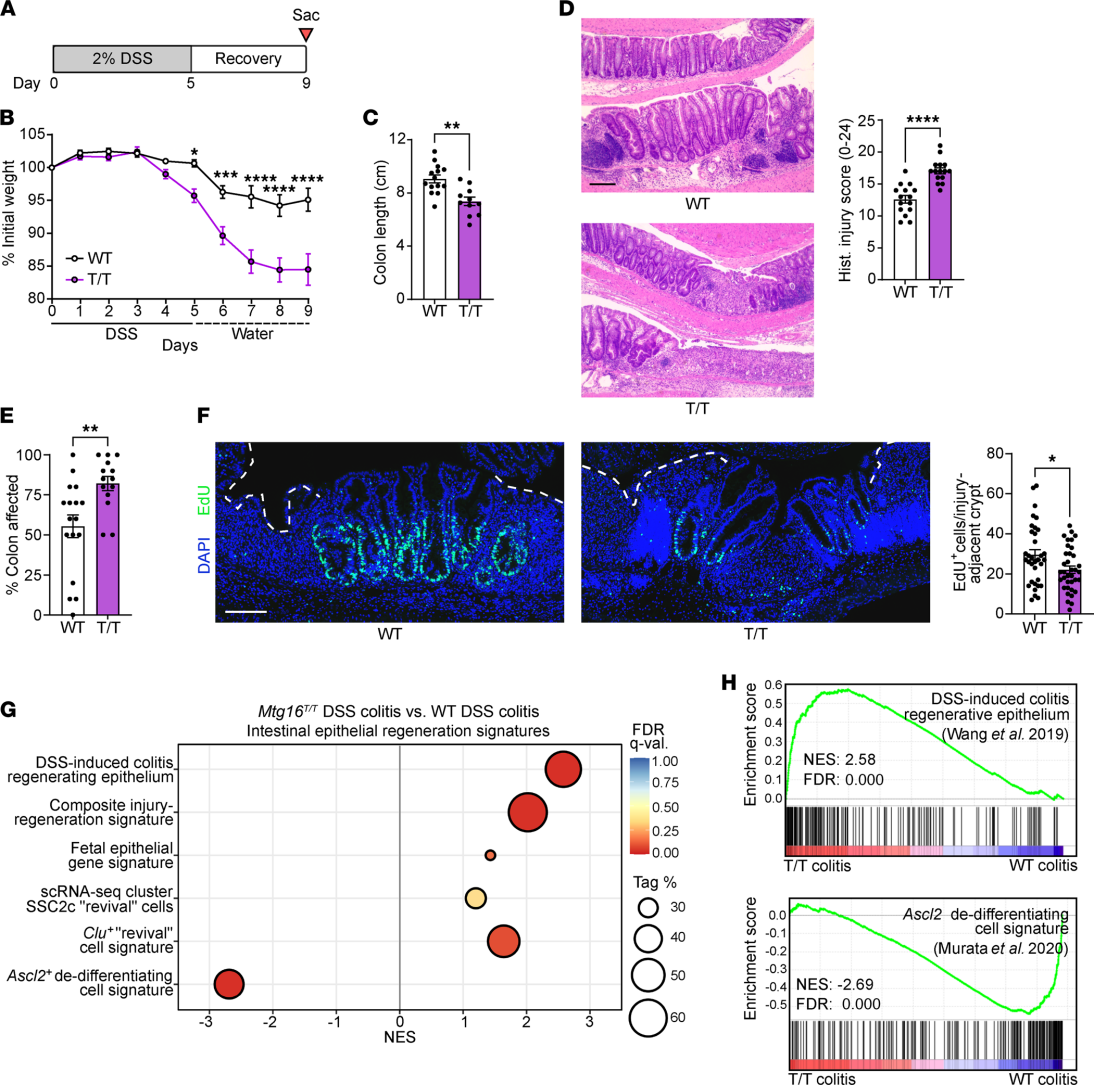
MTG16-mediated colonic epithelial regeneration is dependent on its repression of E protein transcription factor–mediated transcription. (**A**) Schematic of the DSS injury-regeneration model. (**B**–**D**) Colitis severity in *Mtg16^T/T^* (T/T) versus WT mice evaluated using (**B**) weight loss, (**C**) colon length, and (**D**) histologic injury (scoring system described in Supplemental Table 2). Representative images at left. Scale bar = 200 μm. (**E**) Percentage of colon with histologic findings, estimated from the distal end, as a measure of proximal extent of disease. (**B**–**E**) Data are combined from 2 independent experiments (*n* = 15 WT, 16 *Mtg16^T/T^*). (**F**) EdU^+^ cells in injury-adjacent, regenerating crypts in colons from mice injected with EdU 1 hour prior to sacrifice on day 9 (*n* = 30–50 crypts from *n* = 5 mice). Dashed white line indicates ulcerated epithelium. Scale bar = 100 μm. (**B**–**F**) *P *<* 0.05, **P *<* 0.01, ***P *<* 0.001, ****P *<* 0.0001 by (**B**) repeated measures 2-way ANOVA followed by Holm-Šidák multiple-comparison tests or (**C**–**F**) 2-tailed Mann-Whitney test. (**G** and **H**) GSEA of distal *Mtg16^T/T^* versus WT colon crypt isolates following DSS injury-regeneration (*n* = 3) (gene sets described in Supplemental Tables 3 and 4 and citations in the supplement). Tag %, the percentage of gene hits before (for positive ES) or after (for negative ES) the peak in the running ES, indicating the percentage of genes contributing to the ES. FDR *q* < 0.05 is considered significant.

To test whether MTG16-dependent regeneration occurs via an E protein–dependent mechanism, we next analyzed colonic epithelial regeneration following DSS-induced injury (Figure 6A) in *Mtg16^T/T^* versus WT mice. Like *Mtg16^–/–^* mice, *Mtg16^T/T^* mice developed worse colitis and poor regeneration measured by increased weight loss (Figure 6B), decreased colon length (Figure 6C), and increased histologic injury-regeneration score (Figure 6D and Supplemental Table 2) compared with WT. *Mtg16^T/T^* mice also exhibited increased extent of injury along the length of the *Mtg16^T/T^* colon (Figure 6E). Next, we injected mice with 5-ethynyl-2′-deoxyuridine (EdU) 1 hour prior to sacrifice following DSS injury and regeneration and found that *Mtg16^T/T^* mice had fewer S-phase cells in injury-adjacent crypts (Figure 6F), indicating a proliferation defect in the crypts that actively regenerate the ulcerated colonic epithelium following DSS-induced colitis (68). Finally, like we observed in our *Mtg16^–/–^* analyses, RNA-Seq of *Mtg16^T/T^* distal colon crypt epithelial isolates following DSS injury and regeneration was de-enriched for the ASCL2-mediated dedifferentiation signature despite the enrichment of other gene signatures associated with regeneration (Figure 6, G and H, and Supplemental Tables 3 and 4). These data suggest that the effect of MTG16 loss on ASCL2-mediated colonic epithelial regeneration is directly or indirectly due to increased E protein transcription targets.

### MTG16 expression is decreased in dysplasia.

**Figure 7:**
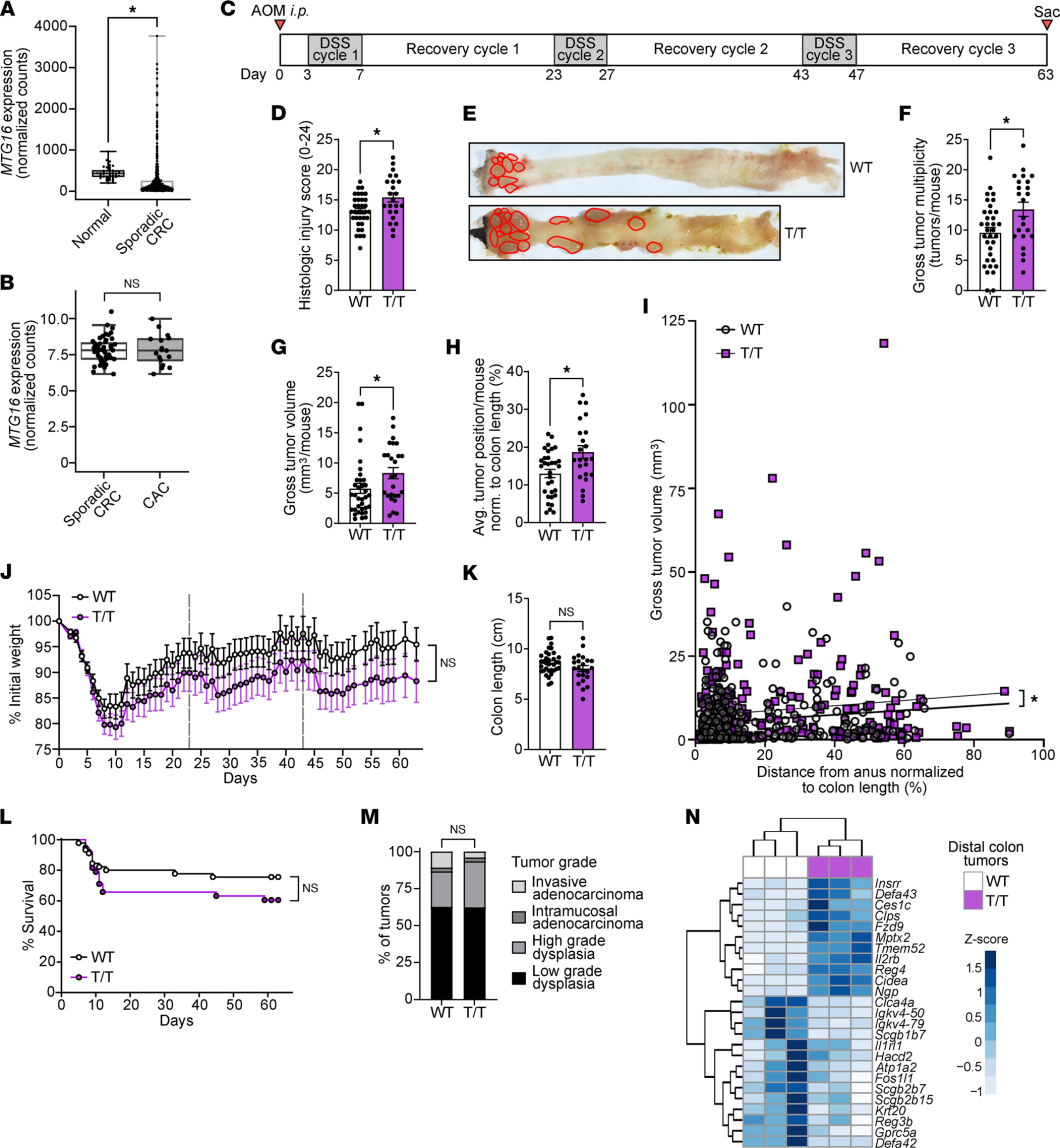
MTG16-mediated protection from tumorigenesis is partially dependent on repression of E protein activity. (**A**) *MTG16* expression in sporadic CRC (*n* = 482) compared with normal colon tissue (*n* = 41) by DESeq2 of TCGA raw counts generated by Rahman et al. (69). The lines within each box represent the mean, the bounds of the boxes represent the 25th to 75th percentiles, and the bounds of the whiskers represent the range of the data. All individual data points are shown. *P_adj_ < 0.05 by DESeq2. (**B**) *MTG16* expression in sporadic CRC (*n* = 47) and CAC (*n* = 17) tumors. *MTG16* expression was normalized by DESeq2 and limma batch correction. The box extends from the lower to upper quartile values of the data, with a line at the median. The lower whisker is at the lowest datum above Q1 – 1.5*(Q3–Q1), and the upper whisker at the highest datum below Q3 + 1.5*(Q3–Q1), where Q1 and Q3 are the first and third quartiles. (**C**) Schematic of the AOM/DSS model in which mice are injected i.p. with AOM followed by 3 cycles of DSS-induced injury and recovery prior to sacrifice. (**D**–**G**) CAC severity in AOM/DSS-treated *Mtg16^T/T^* (T/T) versus WT mice assessed by (**D**) histologic injury-regeneration score (scoring system described in Supplemental Table 2), (**E** and **F**) tumor multiplicity, and (**G**) average tumor volume per mouse. (**H**) Average tumor position normalized to colon length (distance from anus/colon length × 100%) per mouse. (**I**) Individual tumors graphed by size versus distance from anus normalized to colon length. (**J**) Weight loss compared using repeated measures 2-way ANOVA (*n* = 26 WT, 25 *Mtg16^T/T^*, representative of 2 independent experiments). (**K**) Colon length measured at sacrifice. (**L**) Survival curves compared using log-rank (Mantel-Cox) test. (**M**) Percentage of tumors in each grade of dysplasia, evaluated by a pathologist blinded to genotype and compared by χ^2^ test. (**D**–**F**, **G**–**I**, and **K**–**M**) Data are pooled from 2 independent experiments with *n* = 35 WT, 26 *Mtg16^T/T^* remaining at sacrifice. *P *<* 0.05 by (**D**, **F**–**H**, and **K**) 2-tailed Mann-Whitney test or (**I**) least-squares regression. (**N**) Unsupervised hierarchical clustering of differentially expressed genes in *Mtg16^T/T^* versus WT (*n* = 3) distal colon tumors by RNA-Seq. Gene expression in individual samples is displayed by *z* score (normalized count).

Because *MTG16* expression was upregulated in IBD, we wondered whether *MTG16* expression would be increased in IBD-driven colon cancer (CAC) compared with sporadic colorectal cancer (CRC). We previously observed decreased *MTG16* expression in sporadic CRC and CAC compared with normal colon tissue (18) but had not directly compared expression levels of *MTG16* between these different neoplastic origins. To do so, we first confirmed our previous findings in sporadic CRC using DESeq2 on raw counts generated by Rahman et al. (69) from The Cancer Genome Atlas (TCGA) (70) (Figure 7A). We then analyzed *MTG16* expression in bulk RNA-Seq of CRC and CAC tumors (71) and found no significant difference in *MTG16* expression (Figure 7B), indicating that *MTG16* expression is reduced in colon dysplasia regardless of whether it is driven by IBD.

### MTG16-mediated protection from CAC is partially dependent on MTG-mediated repression of E protein transcription factors.

We previously showed that *Mtg16^–/–^* mice have increased mortality, tumor burden, tumor grade, and tumor cell proliferation and apoptosis, as well as alterations in the intratumor immune environment in AOM/DSS-induced CAC (18). We next tested whether the mechanism driving these phenotypes was E protein dependent by treating *Mtg16^T/T^* mice with AOM/DSS (Figure 7C). Like *Mtg16^–/–^* mice, *Mtg16^T/T^* mice did exhibit increased histologic injury, tumor multiplicity, and tumor size compared with WT (Figure 7, D–G). Interestingly, *Mtg16^T/T^* mice had more tumors and larger tumors in the middle and proximal colon than their WT counterparts (Figure 7, H and I). This was consistent with DSS-induced injury extending further toward the proximal colon (Figure 6E). These data suggest that this phenotype could be driven by increased proximal colon injury. However, unlike *Mtg16^–/–^* mice, *Mtg16^T/T^* mice did not have significantly more weight loss (Figure 7J), colon shortening (Figure 7K), mortality (Figure 7L), or tumor dysplasia (Figure 7M) compared to WT.

We further characterized proliferation, apoptosis, and DNA damage in *Mtg16^T/T^* tumor epithelial cells (Supplemental Figure 8, A–C) and the *Mtg16^T/T^* intratumor immune cell environment by immunofluorescence staining (Supplemental Figure 8, D–G). Unlike our previous characterization of *Mtg16^–/–^* tumors, our data demonstrated no increase in tumor epithelial cell proliferation (Supplemental Figure 8A), a decrease (instead of increase) in tumor epithelial cell DNA damage (Supplemental Figure 8C), no difference in F4/80^+^ intratumor macrophages (Supplemental Figure 8E), a subtle decrease in tumor-infiltrating CD3^+^ T cells (Supplemental Figure 8F), and no decrease in infiltrating B220^+^ B cells (Supplemental Figure 8G). We did not observe a difference in intratumor Ly6B.2^+^ cells (Supplemental Figure 8D), which we did not evaluate in the *Mtg16^–/–^* AOM/DSS mouse study. We did, however, observe a subtle increase in apoptotic epithelial cells per tumor (Supplemental Figure 8B), similar to our prior findings in *Mtg16^–/–^* tumors (18). The epithelial phenotypes in Supplemental Figure 8, A–C, were recapitulated in tumoroids derived from distal, but not proximal, WT and *Mtg16^T/T^* tumors (Supplemental Figure 9).

Finally, we performed RNA-Seq on distal colon tumors from littermate- and cage-matched WT and *Mtg16^T/T^* mice to test whether *Mtg16^T/T^* AOM/DSS tumors would exhibit the increased canonical WNT signaling tone that we previously demonstrated in *Mtg16^–/–^* AOM/DSS tumors (18). On the contrary, RNA-Seq demonstrated differential expression of only 1 WNT-related gene, the WNT receptor *Fzd9* (Figure 7N), and GSEA did not demonstrate enrichment of the canonical WNT signaling pathway (unpublished observation). We did observe increased expression of enteroendocrine-associated genes, potentially attributable to the epithelial phenotype at baseline (Figure 7N). GSEA using the oncogenic signatures found in the MSigDB demonstrated deranged cell cycle control and KRAS signaling, though many conflicting gene sets were enriched (unpublished observation). Overall, these data indicate that *Mtg16^T/T^* mice recapitulate few *Mtg16^–/–^* AOM/DSS phenotypes, suggesting that MTG16 protects the colonic epithelium from tumorigenesis through additional mechanisms.

## Discussion

In this study, we investigated the mechanism underlying context-specific functions of the transcriptional corepressor MTG16. We identify what we believe are previously unappreciated roles for MTG16 as a critical regulator of colonic secretory cell differentiation and active colon regeneration. To define the mechanism underlying these phenotypes, we utilized a mouse model with a single point mutation in MTG16, MTG16^P209T^, that attenuates direct binding between MTG16 and the E proteins E2A and HEB (52). *Mtg16^T/T^* mice homozygous for this MTG16:E protein uncoupling mutation largely recapitulated *Mtg16^–/–^* lineage allocation and regeneration phenotypes, though the effect of MTG16 loss in inflammation-driven dysplasia appears to partially be due to increased E protein activity. Thus, these data indicate potentially new roles for MTG16:E protein complexes in colonic epithelial homeostasis, regeneration, and tumorigenesis.

First, we demonstrated a potentially novel role for MTG16 in colonic epithelial homeostasis. MTG16 has been shown to repress stem, goblet, and enteroendocrine cell genes to drive enterocyte differentiation in the SI (16). However, in accordance with numerous studies demonstrating important differences between SI and colon biology (2, 36, 72–75), *Mtg16^–/–^* colonic epithelial isolates did not exhibit increased stem cell transcriptional signatures or an overall decrease in secretory genes. Instead, we observed specific upregulation of E protein transcriptional signatures and the enteroendocrine lineage. This could be due to the fact that MTG16 repression targets, and its effects on them, vary in different tissues and cell types due to expression gradients of its binding partners (14). Another potential explanation for the difference between SI and colonic phenotypes is that SI secretory progenitor cells that express *Neurog3*, the class II bHLH transcription factor required for enteroendocrine cell differentiation (43, 44), may still differentiate into goblet and Paneth cells, while colonic secretory progenitor cells that express *Neurog3* are fully committed to the enteroendocrine lineage in the colon (46, 73). Compared with WT, *Mtg16^–/–^* colon crypts demonstrated increased expression of *Neurog3* by RNA-Seq, increased *Neurog3*^+^ secretory progenitor cells per colon crypt by RNAscope, and significant enrichment (FDR *q* < 0.05) of gene sets representing all enteroendocrine lineage cells starting at the *Neurog3*^+^ enteroendocrine progenitor stage. Importantly, expression of *Neurog3* is transient and does not continue as enteroendocrine cells progress through differentiation (45). Thus, we believe the transcriptomic changes we observed by bulk RNA-Seq are not simply a reflection of the phenotypic decrease in differentiated enteroendocrine cells we observed by IHC for SYP and immunofluorescence staining for CHGA.

Next, we mechanistically probed the extent to which MTG16:E protein interactions drive MTG16-associated lineage allocation in the colon using a mouse model homozygous for an amino acid substitution in MTG16 (MTG16^P209T^) (52). Strikingly, with this single amino acid change, *Mtg16^T/T^* mice recapitulated *Mtg16^–/–^* colonic secretory phenotypes, including increased enteroendocrine cells, increased *Neurog3* expression by RNA-Seq, increased *Neurog3*^+^ progenitors, enrichment of gene sets representing all enteroendocrine lineage cells starting at the *Neurog3*^+^ enteroendocrine progenitor stage, and decreased goblet cells. Analysis of a publicly available SI ChIP-Seq data set (16) indicated MTG16 occupancy of an E box–rich region at the *Neurog3* promoter. From these data, we believe that MTG16 prevents the differentiation of secretory progenitor cells toward the enteroendocrine cell lineage, enabling differentiation of the goblet cell lineage, by repressing E protein oligomers at the *Neurog3* promoter. MTG16 could be repressing E protein homo-oligomers at this locus, as E2A homodimers have recently been found to promote neural differentiation and increased *Neurog3* expression and in pluripotent cells, even under nonpermissive conditions (31). MTG16 could also be repressing E protein:class II bHLH transcription factor hetero-oligomers activating transcription of the *Neurog3* locus. One class II bHLH transcription factor known to bind to the *Neurog3* promoter is atonal bHLH transcription factor 1 (ATOH1) (76). However, MTG16 has been shown to bind ATOH1 transcriptional targets involved in the differentiation of multiple secretory lineages, including *Spdef*, the transcription factor known for initiating goblet cell differentiation (16). It is possible that the enteroendocrine-promoting effect of increased E protein activity is amplified following increased induction of *Neurog3*. NEUROG3 induces transcription of *Neurod1*, the next class II bHLH transcription factor in the enteroendocrine transcription factor cascade (45, 77), probably through heterodimerization with E proteins, which has been demonstrated in pancreatic endocrine cells (78, 79). In turn, NEUROD1: E protein complexes induce transcription of other enteroendocrine transcription factors and hormones (50, 77). On the contrary, SPDEF is not a bHLH transcription factor, has not been shown to bind to E proteins, and has not been shown to be repressed by MTG16. Additionally, complex regulatory relationships are at play in these transcription factor cascades, as *Mtg16* itself has been shown to be a transcriptional target of E proteins in the hematopoietic system (1) and ATOH1 in both SI and colon (29).

Another transcription factor recently shown to be involved in SI and colonic secretory lineage allocation is retinoic acid receptor alpha (RARα). Jijon et al. demonstrated that *Vil1-Cre* *RARα^fl/fl^* mice develop increased goblet cells in the SI and decreased enteroendocrine cells in both the SI and colon (80). Recently, Steinauer et al. demonstrated that MTG16 regulates histone acetylation and chromatin accessibility of myeloid-specific enhancers to reduce expression of RARα target genes in human acute myeloid leukemia cells (81). This mechanism could explain increased colonic enteroendocrine cells in the setting of MTG16 loss but does not entirely explain the colonic goblet cell phenotype observed in our study. Additionally, GSEA of our bulk transcriptomic data did not reveal upregulation of RARα target genes or the RAR pathway in *Mtg16^–/–^* or *Mtg16^T/T^* colonic epithelial isolates (unpublished observation). Studies have shown that RARα antagonizes E protein activity by inducing transcription of *ID1* and *ID2* in human acute promyelocytic leukemia cells treated with all-*trans*-retinoic acid (82) and *ID1* and *ID3* gene expression in normal human keratinocytes (83). Thus, it is possible that downstream effects of the RAR pathway counteract the effects of MTG16:E protein uncoupling in our bulk RNA-Seq data. Finally, another non–E protein–mediated pathway involved in secretory lineage allocation is the noncanonical, β-catenin–independent WNT/planar cell polarity (PCP) pathway, which may enable enteroendocrine cells to differentiate directly from “unipotentially primed” intestinal stem cells (84). The WNT/PCP pathway was mildly enriched (FDR *q* value = 0.11) in *Mtg16^–/–^*, but not *Mtg16^T/T^*, colon crypts. This could be responsible for the slight difference in magnitude between *Mtg16^–/–^* and *Mtg16^T/T^* phenotypes. These are interesting areas of further investigation.

An area of considerable interest in gastrointestinal biology is the relationship between epithelial differentiation and regeneration (4). Although it was long thought that dedicated *Bmi1*^+^ or *mTert*^+^ “reserve” stem cells at the +4/+5 position replenished the stem cell compartment in intestinal epithelial regeneration, recent data indicate that these cells are enteroendocrine cells that can dedifferentiate into stem cells following injury (47). Recently, “committed” progenitors from both the secretory and absorptive lineages were demonstrated to share this ability through induction of ASCL2 (2). In this study, *Ascl2*^+^ dedifferentiating cells were isolated from the colonic epithelium following CBC ablation (2). Interestingly, these cells were not enriched for *Clu* or fetal epithelial genes (2), which were previously thought to enhance intestinal epithelial response to injury (63–67). *Mtg16* was one of the few genes upregulated in these regenerating cells, and ChIP-Seq demonstrated an ASCL2 peak at a second TSS in the *Mtg16* locus (2). Demonstrating relevance to DSS-induced colon regeneration, both *Mtg16* and the *Ascl2^+^* regenerating cell signature were upregulated in regenerating WT colon epithelium compared with baseline. *MTG16* was also upregulated in multiple IBD patient cohorts and even decreased with remission, during which the colon epithelium is closer to homeostatic renewal. Finally, RNA-Seq and GSEA of *Mtg16^–/–^* and *Mtg16^T/T^* colonic epithelial isolates following DSS-induced injury demonstrated enrichment of “revival” stem cell signatures and fetal epithelial signatures but de-enrichment of the *Ascl2^+^* regenerating cell signature. These data indicate an active, E protein–mediated role for MTG16 in colon regeneration and provide a potential explanation for defective colonic epithelial regeneration in *Mtg16^–/–^* mice despite an apparent baseline enrichment of *Bmi1*^+^ and *mTert*^+^ “reserve” stem cells. Future work is necessary to determine which E protein complexes and gene targets are repressed by MTG16 in *Ascl2^+^* dedifferentiating cells. One possibility is that ASCL2-induced *Mtg16* expression forms a negative feedback loop in which MTG16 attenuates stemness so that not all cells in the crypt dedifferentiate into *Lgr5*^+^ stem cells. This is supported by our previous work demonstrating that MTG16 negatively regulates major transcription factor effectors of the canonical WNT signaling pathway in the hematopoietic system (15), although we did not observe canonical WNT pathway activation by transcriptomic analyses of the homeostatic or regenerating *Mtg16^–/–^* colonic epithelia. Instead, ASCL2 could be inducing *Mtg16* expression to prevent dedifferentiating cells from expressing *Neurog3*, reversing course, and differentiating toward the enteroendocrine lineage. Notably, although ASCL2 is a class II bHLH transcription factor, our study does not attempt to address whether MTG16 inhibits ASCL2:E protein complexes downstream of *Ascl2*-induced Mtg16 expression. These are interesting areas of future investigation.

Finally, we tested the role of MTG16:E protein interactions in inflammatory carcinogenesis. We previously showed that *Mtg16^–/–^* mice develop greater tumor burden than their WT counterparts with AOM/DSS treatment, and *Mtg16^–/–^* tumors exhibit increased WNT tone (18). Although we did observe increased histologic injury, tumor burden, and tumor size in *Mtg16^T/T^* mice, *Mtg16^T/T^* tumors did not recapitulate the higher grade of dysplasia observed in *Mtg16^–/–^* tumors or enrichment in the WNT signaling pathway. Thus, disabling one known function of MTG16 was not sufficient to fully replicate the *Mtg16^–/–^* phenotype. Indeed, our group recently showed that the effects of MTG16 loss on CAC development are dependent on Kaiso (ZBTB33) (85). Additionally, E proteins have been shown to be both antitumorigenic and protumorigenic in colon cancer via epithelium-intrinsic and -extrinsic effects, including attenuation of the canonical WNT signaling pathway (86–88). Future studies are necessary to determine additional MTG16 binding partners and repression targets responsible for suppressing carcinogenesis.

In conclusion, we discovered key functions of MTG16 in colonic secretory lineage allocation and regeneration following colitis dependent on its repression of E proteins. Confirming translational relevance, we determined that *MTG16* is upregulated in patients with active IBD, reduced with restitution, and decreased in dysplasia. Thus, *MTG16* may be a candidate biomarker for disease activity or a target to modulate differentiation and regeneration.

## Methods

### Animal models.

*Mtg16^–/–^* mice were previously generated and backcrossed to a C57BL/6J background (89). *Mtg16*^P209T^ mutant mice were generated by the Vanderbilt Genome Editing Resource (VGER) using CRISPR/Cas9-mediated dsDNA cleavage followed by homology-directed repair. Briefly, 50 ng/μL guide RNA (5′ TTTGTTATCCCTTTTCTGAAGG 3′), 200 ng/μL of 119 bp single-stranded donor DNA (5′ GTTTCATGCCAAGCTCCAGGAAGCCACCAACTTTCCACTGAGGCCGTTTGTTAT-AACTTTTCTGAAAGTAAGCGAAAGCAGCACCTTCCAGGCAACAGGGATGGTGTACACAAAGGCCC 3′), and 100 ng/μL Cas9 mRNA were microinjected into the cytoplasm of C57BL/6J mouse embryos. Embryos were implanted into the oviduct of pseudopregnant female mice. The resulting pups were screened for successful editing by PCR. PCR for the WT *Mtg16* allele was performed using the primers SWH1432 (5′ AGCACCTGCCCCCGGCGTGCG 3′) and SWH1438 (5′ TTGCCTGGAAGGTGCTGCTTTCGCTTACCTTCAGAAAAGGG 3′). PCR for the *Mtg16*^P209T^ mutant allele was performed using the primers SWH1432 and SWH1435 (5′ TTGCCTGGAAGGTGCTGCTTTCGCTTACTTTCAGAAAAGTT 3′). Both reactions were performed using the following thermal cycle: 95°C for 5 minutes initial denaturation; 35 cycles of 95°C for 30 seconds, 61°C for 1 minute, and 72°C for 5 minutes; and 72°C for 10 minutes final extension (representative reactions in Supplemental Figure 7, A and B; full, uncropped gels available in online supplemental material). On-target edits in potential founders were validated by genomic sequencing of the donor DNA sequence and at least 200 bp of flanking genomic DNA. Founders were crossed with WT C57BL/6J mice to minimize any remaining off-target effects. Expression of full-length MTG16 (MTG16^P209T^) in *Mtg16^T/T^* mice was confirmed by immunoblot (Supplemental Methods and Supplemental Figure 7C; full, uncropped gels available in online supplemental material). Littermate-matched WT and *Mtg16^–/–^* and WT and homozygous *Mtg16*^P209T^ mutant (*Mtg16^T/T^*) mice were bred in independent mouse colonies using heterozygous × heterozygous breeding schemes, housed in the same facility with a 12-hour light/12-hour dark cycle, and provided with standard rodent chow (5L0D, LabDiet) ad libitum. Approximately equal numbers of 8- to 12-week-old male and female mice were used in each experiment.

### scRNA-Seq.

InDrop scRNA-Seq and analysis were performed on 2 independent (discovery and validation) cohorts of human colon samples as previously described (90). For the Colorectal Molecular Atlas Project (discovery) cohort, cells were encapsulated using a modified inDrop platform (91). Libraries were prepared using the TruDrop protocol ll (92) and sequenced to a target of 150 million reads (90). Results were validated using independent data from the Broad Institute (validation cohort) (90). *MTG16* expression was queried in normal human colon cells identified in the discovery and validation data sets, which are publicly available through the Human Tumor Atlas Network (https://data.humantumoratlas.org/) (93). *Mtg16* expression was queried from murine scRNA-Seq data generated from WT colon crypts using the inDrop platform (1CellBio) as previously described (34, 35, 94). These data (GSE145830, GSE114044) are available in the National Center for Biotechnology Information’s Gene Expression Omnibus (NCBI GEO) (95, 96).

### RNAscope.

RNAscope, high-resolution RNA in situ hybridization (97), was performed on FFPE sections using the RNAscope Multiplex Fluorescent V2 Assay (323100, ACDBio). Slides were boiled in Target Retrieval Reagents for 15 minutes and treated with Protease IV for 30 minutes. Probes were specific for Mm-*Cbfa2t3* (43601-C2, ACDBio), Mm-*Muc2* (315451, ACDBio), and Mm-*Neurog3* (422401, ACDBio); 1:750 TSA Cy3 (NEL704A001KT, PerkinElmer) and TSA Cy5 (NEL75A001KT, PerkinElmer) were used for probe visualization.

### Chromogenic and immunofluorescence IHC.

SI and colon were dissected, Swiss-rolled, and fixed in 10% neutral-buffered formalin (NBF) for 24 hours at room temperature (RT). The Vanderbilt Translational Pathology Shared Resource (TPSR) paraffin-embedded the tissue and performed H&E and PAS staining. Chromogenic and immunofluorescence IHC were performed as previously described (85, 98). Briefly, 5 μm FFPE sections were deparaffinized, rehydrated in graded ethanol concentrations, and permeabilized using TBS-T (Tris-buffered saline with 0.05% Tween 20), followed by antibody-specific antigen retrieval (Supplemental Table 1). Next, for chromogenic IHC, endogenous peroxidases were quenched in 0.03% H_2_O_2_ with sodium azide for 5 minutes. Slides were then incubated in primary antibody (Supplemental Table 1) overnight (O/N) at RT. Detection was performed by 30 minutes’ incubation with Dako Envision+ System HRP-Labeled Polymers (K4003, Agilent) followed by a 5-minute incubation with DAB. For immunofluorescence staining, slides were blocked in 5% normal goat serum (01-620-1, Thermo Fisher Scientific), incubated in primary antibody (Supplemental Table 1) O/N at 4°C, washed in TBS-T, and incubated in the appropriate secondary antibodies (A-11011, A-11077, A-21131, Invitrogen) for 2 hours at RT. EdU was visualized using the Click-iT EdU Cell Proliferation Kit for Imaging (C10037, Thermo Fisher Scientific). Nuclear counterstaining was performed using ProLong Gold Antifade Mountant with DAPI (P36931, Thermo Fisher Scientific).

### Slide imaging and quantification.

Slides were imaged using a Nikon Eclipse E800 microscope with NIS-Elements Basic Research Software or by the Vanderbilt University Medical Center Digital Histology Shared Resource (DHSR). For chromogenic IHC, whole slides were imaged by the DHSR using a Leica SCN400 Slide Scanner (Leica Biosystems). For fluorescence staining, whole slides were imaged by the DHSR using an Aperio Versa 200 automated slide scanner (Leica Biosystems) and visualized using Aperio ImageScope (v12.4.3) (Leica Biosystems). Quantification was performed, according to a protocol blinded to genotype, by counting positive cells in well-oriented crypts or crypt-villus units.

### Colon crypt isolation.

Murine colon was harvested and splayed longitudinally. The proximal (5 cm from cecum) and distal (5 cm from anus) colon were washed in PBS without calcium or magnesium and minced. Tissue fragments were incubated in chelation buffer (2 mM EDTA in PBS without calcium or magnesium) with nutation for 90 minutes at 4°C and washed with PBS followed by 2-minute cycles of gentle shaking in shaking buffer (43.3 mM sucrose and 54.9 mM sorbitol in PBS without calcium or magnesium) to free colon crypts. Intact crypts were collected by centrifugation at 150*g*, 4°C, for 12 minutes for RNA isolation and bulk RNA-Seq as described below.

### DSS-induced colitis.

Bedding was mixed between cages to normalize the microbiome 2 weeks prior to experiments. Water deprivation caps were placed to accustom mice to drinking from water bottles 1 week prior to experiments. Water bottles were filled with 2% (w/v) DSS (DS1004, Gojira Fine Chemicals) for 5 days and then replaced with water for 4 days of recovery prior to sacrifice. At sacrifice, colons were dissected, Swiss-rolled, fixed in 10% NBF, and processed by the Vanderbilt TPSR. H&E slides were scored for histologic injury and regeneration using the scoring system described in Supplemental Table 2 by a gastrointestinal pathologist blinded to genotype. Distal colon crypts were collected as described above for RNA isolation and RNA-Seq as described below. For EdU experiments, mice were i.p. injected with 50 mg/kg EdU (NE08701, Biosynth Carbosynth) in PBS 1 hour prior to sacrifice. Mice were weighed daily throughout all experiments.

### Inflammatory carcinogenesis.

Mice were prepared as described above and injected i.p. with 10 mg/kg AOM (A5486, MilliporeSigma) followed by three 4-day cycles of 3% (w/v) DSS (DB001, TdB Labs), each followed by 16 days of recovery. Mice were weighed daily. At sacrifice, colons were dissected, flushed with PBS, splayed longitudinally, and imaged using a Nikon SMZ1270 dissecting microscope. Tumors were identified by vascular, nondimpled appearance from these images in conjunction with direct visualization and measured macroscopically in 2 perpendicular dimensions using calipers. Tumor volume was calculated using the previously validated formula (W^2^ × L)/2, where width (W) was the shorter caliper measurement, and length (L) was the longer caliper measurement (99). Representative tumors from each colon were dissected, incubated in RNA*later* (R0901, MilliporeSigma) at 4°C for 24 hours, and stored at –80°C. Additional representative tumors were used to generate tumoroids (Supplemental Methods). Histologic injury and regeneration were assessed (using the scoring system in Supplemental Table 2). Grade of dysplasia was evaluated as previously described (100) by a gastrointestinal pathologist blinded to genotype.

### RNA isolation, bulk RNA-Seq, and analysis.

Murine colon crypts were collected as described above followed by resuspension in TRIzol Reagent (15596018, Invitrogen) and homogenization by 5x shearing through a 25G needle. Murine tumors were mechanically homogenized in TRIzol using an electronic homogenizer. Samples were centrifuged for 10 minutes at 16,200*g*, 4°C, to remove insoluble material. Cleared homogenate was chloroform extracted. RNA was isolated from the aqueous layer using the RNeasy Mini Kit (74106, QIAGEN) with on-column DNase treatment (79254, QIAGEN). Library preparation and paired-end 150 bp RNA-Seq using the Illumina NovaSeq6000 were performed by Vanderbilt Technologies for Advanced Genomics. Raw reads in FASTQ format were trimmed with fastp (v0.20.0) with default parameters (101). Quantification was performed using Salmon (v1.3.0) (102) against a decoy transcriptome generated from *Mus musculus* GENCODE (v21) (103). Further analysis was performed in R (v3.6.3) as described previously (104). Briefly, quantification files were imported with tximeta (v1.4.5) (105). Genes with counts ≤ 1 across all samples were omitted. Differential expression analysis (DEA) was performed on raw transcript counts using DESeq2 (v1.26.0) (106) and annotated with AnnotationDbi (v1.46.1) (107). GSEA (108) was performed on DESeq2-normalized transcript counts using the GSEA software (v4.1.0) with 1000 gene permutations, gene set randomization, and gene sets found in the MSigDB (v7.2) (51) or derived from the literature (Supplemental Tables 3 and 4). RNA-Seq of human CRC and CAC samples collected from 9 regional hospitals in Finland was performed and analyzed as described previously (71) (Supplemental Methods). RNA-Seq data are publicly available in the NCBI’s GEO (accession GSE201768).

### In silico analyses of publicly available data sets.

Raw and processed ChIP-Seq data from Baulies et al. (16) (GSE124186) and Murata et al. (2) (GSE130822) were retrieved from the GEO (95). Data were visualized and E box (5′-CATNNG) sequences were annotated using the Broad Institute Integrated Genomics Viewer (v2.9.1) (109). Metadata and SRR files from the PROTECT study (58, 59) (GEO GSE109142) were downloaded using the NCBI SRA Toolkit and processed as described above for murine samples, with reads quantified against a decoy-aware transcriptome generated from the GENCODE human reference genome (v29) (103). DEA was performed on raw counts using DESeq2 (v1.26.0) (106). Pairwise DEA was also performed on raw counts from RNA-Seq of colon biopsies from adults with UC (60) (GSE128682) and raw counts generated from TCGA (70) by Rahman et al. (69) (GSE62944) using DESeq2 (v1.33.1) (106). Microarray data from patients with IBD (61) (GSE9686) were analyzed using limma in Geo2R (96). The *Tabula Muris* (42) was queried for *Mtg16* expression using its web interface (https://tabula-muris.ds.czbiohub.org/).

### Statistics.

Statistical analyses of human CAC and sporadic CRC samples were performed using limma (v3.34.9) (110). Statistical analyses of all other bulk RNA-Seq were performed using DESeq2 (106). Statistical analyses of microarray data were performed using limma in Geo2R (97). *P*_adj_ < 0.05 was considered significant. Statistical analyses for GSEA were performed using the GSEA program (v4.1.0). FDR (*q* value) < 0.05 was considered significant. All other statistical analyses (nonparametric, 2-tailed comparisons between 2 or more groups) were performed in GraphPad Prism (v9.0.1) as indicated in each figure legend. Data are displayed as arithmetic mean ± SEM unless otherwise noted. *P* < 0.05 was considered significant.

### Study approval.

All animal experiments were carried out in accordance with protocols approved by the Vanderbilt IACUC (Nashville, Tennessee, USA). Human CRC and CAC studies (71) were approved by the Ethics Committee of the Hospital District of Helsinki (Helsinki, Finland) and the Finnish Institute for Health and Welfare (Helsinki, Finland).

## Author contributions

REB designed experiments, performed experiments, and analyzed data. SAA, KMB, CTJ, JMP, and AHG performed experiments and analyzed data. JJ and REB designed and performed bulk RNA-Seq data analysis, respectively. KSL, BC, and PNV designed, collected, and analyzed human and murine colon scRNA-Seq. FR validated antibodies for and performed IHC staining. MBP and MKW performed histologic analyses and provided expertise in gastrointestinal pathology. JJ, REB, and KP analyzed RNA-Seq from human tumors. CSW, SPS, SWH, KSL, JAG, KRS, and YAC provided expertise regarding experimental models and experimental design. REB wrote the manuscript. All authors edited and approved the manuscript.

## Supplementary Material

Supplemental data

Supplemental table 4

## Figures and Tables

**Table 1:**
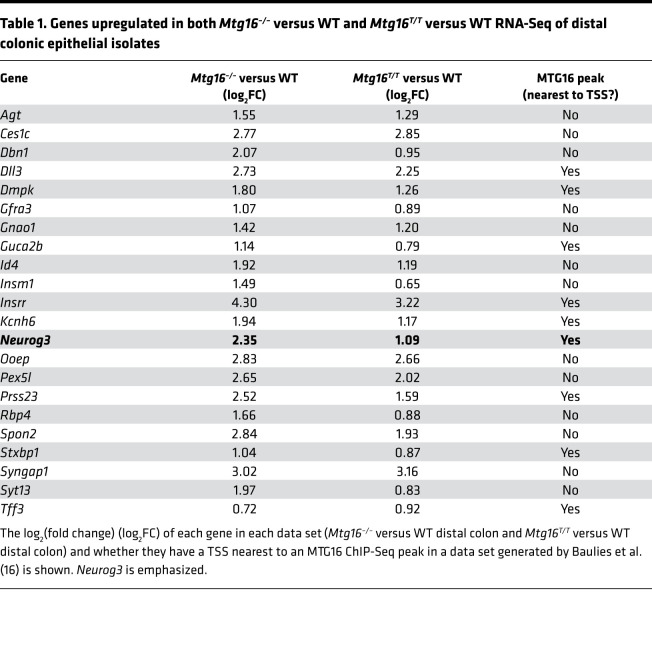
Genes upregulated in both *Mtg16^–/–^* versus WT and *Mtg16^T/T^* versus WT RNA-Seq of distal colonic epithelial isolates

## Version 1 05/03/2022

In-Press Preview

## Version 2 05/23/2022

Electronic publication
